# Comparison of light gradient boosting and logistic regression for interactomic hub genes in *Porphyromonas gingivalis* and *Fusobacterium nucleatum*-induced periodontitis with Alzheimer's disease

**DOI:** 10.3389/froh.2025.1463458

**Published:** 2025-03-04

**Authors:** Pradeep Kumar Yadalam, Shubhangini Chatterjee, Prabhu Manickam Natarajan, Carlos M. Ardila

**Affiliations:** ^1^ Department of Periodontics, Saveetha Dental College, Saveetha Institute of Medical and Technical Sciences, Saveetha University, Chennai, India; ^2^ Department of Clinical Sciences, Center of Medical and Bio-allied Health and Research, College of Dentistry, Ajman University, Ajman, United Arab Emirates; ^3^ Department of Basic Sciences, Faculty of Dentistry, Universidad de Antioquia U de A, Medellín, Colombia; ^4^ Biomedical Stomatology Research Group, Universidad de Antioquia U de A, Medellín, Colombia

**Keywords:** periodontal disease, Alzheimer's disease, light gradient boosting, interactome, hub genes, *Porphyromonas gingivalis*, *Aggregatibacter actinomycetemcomitans*, *Fusobacterium nucleatum*

## Abstract

**Introduction:**

*Porphyromonas gingivalis* and Treponema species have been found to invade the central nervous system through virulence factors, causing inflammation and influencing the host immune response. *P. gingivalis* interacts with astrocytes, microglia, and neurons, leading to neuroinflammation. *Aggregatibacter actinomycetemcomitans* and *Fusobacterium nucleatum* may also play a role in the development of Alzheimer's disease. Interactomic hub genes, central to protein-protein interaction networks, are vulnerable to perturbations, leading to diseases such as cancer, neurodegenerative disorders, and cardiovascular diseases. Machine learning can identify differentially expressed hub genes in specific conditions or diseases, providing insights into disease mechanisms and developing new therapeutic approaches. This study compares the performance of light gradient boosting and logistic regression in identifying interactomic hub genes in *P. gingivalis* and *F. nucleatum*-induced periodontitis with those in Alzheimer's disease.

**Methods:**

Using the GSE222136 dataset, we analyzed differential gene expression in periodontitis and Alzheimer's disease. The GEO2R tool was used to identify differentially expressed genes under different conditions, providing insights into molecular mechanisms. Bioinformatics tools such as Cytoscape and CytoHubba were employed to create gene expression networks to identify hub genes. Logistic regression and light gradient boosting were used to predict interactomic hub genes, with outliers removed and machine learning algorithms applied.

**Results:**

The data were cross-validated and divided into training and testing segments. The top hub genes identified were TNFRSF9, LZIC, TNFRSF8, SLC45A1, GPR157, and SLC25A33, which are induced by *P. gingivalis* and *F. nucleatum* and are responsible for endothelial dysfunction in brain cells. The accuracy of logistic regression and light gradient boosting was 67% and 60%, respectively.

**Discussion:**

The logistic regression model demonstrated superior accuracy and balance compared to the light gradient boosting model, indicating its potential for future improvements in predicting hub genes in periodontal and Alzheimer's diseases.

## Introduction

Alzheimer's Disease (AD), first identified in 1906 by the German neuropathologist Alois Alzheimer, is a progressive neurological disorder that leads to cognitive and behavioral difficulties, such as memory loss, trouble forming new memories, and challenges with logical thinking and communication (1). This gradual decline significantly affects quality of life and often results in disabilities, anxiety, and depression. Key pathological features of AD include neurofibrillary tangles (NFT) and amyloid-beta (Aβ) plaques (2–5).

Periodontal disease (PD), a chronic condition affecting nearly half of the adult population worldwide (6), has been linked to systemic illnesses such as diabetes, cardiovascular diseases, and rheumatoid arthritis. Recent research suggests that oral dysbiosis in PD may contribute to the development of AD by enabling oral bacteria to reach the brain through cranial nerves or cellular infections. However, the specific pathogens and mechanisms involved in this link remain unclear (7–9).

In periodontitis, dysbiosis triggers excessive inflammation in susceptible individuals, leading to the destruction of the periodontium, which includes the alveolar bone, periodontal ligament, and cementum. Subgingival bacteria, especially those associated with severe periodontitis, play a key role in this process (2, 3).

Certain pathogens, such as *Porphyromonas gingivalis* and *Treponema* species, can invade the central nervous system (CNS) either directly or via their virulence factors (6, 10, 11). These bacteria have been detected in animal models and in tissue samples from AD patients. Emerging evidence suggests that *Aggregatibacter actinomycetemcomitans* and *Fusobacterium nucleatum* may also contribute to AD. Virulence factors like lipopolysaccharide (LPS) and gingipains from *P. gingivalis* can damage tissues, intensify inflammation, and alter the immune response as periodontitis progresses (7, 12, 13). *P. gingivalis* and gingipains interact with astrocytes, microglia, and neurons, causing neuroinflammation by activating microglia and promoting the release of pro-inflammatory molecules. Similarly, *F. nucleatum* has been linked to AD in mice, inducing morphological changes and elevated levels of TNF-α and IL-1β, suggesting its role as a potential risk factor for AD (13, 14).

Interactomic hub genes are central in protein-protein interaction networks, playing a crucial role in cellular regulation. Disruption of these genes can lead to diseases such as cancer, neurodegenerative disorders, and cardiovascular conditions (15, 16). Identifying and targeting these hub genes can help restore normal physiological functions. Machine learning has emerged as a powerful tool for analyzing gene expression, predicting diseases, and identifying drug targets (17, 18). By identifying differentially expressed hub genes, it offers valuable insights into disease mechanisms and supports the development of new therapeutic approaches. It can also enhance diagnostic accuracy and prioritize drug targets, speeding up drug discovery and improving treatment strategies (19).

Predicting hub genes is vital for drug design. This study compares light gradient boosting and logistic regression models to identify interactomic hub genes involved in *P. gingivalis* and *F. nucleatum*-induced periodontitis and their link to Alzheimer's disease.

## Materials and methods

### Gene expression database

We used the dataset GSE222136 from the NCBI Gene Expression Omnibus (GEO) to analyze periodontitis and Alzheimer's disease. This dataset includes nine samples: three controls, three infected with *F. nucleatum*, and three infected with *P. gingivalis*—two periodontal pathogens linked to Alzheimer's disease (20). To validate hub genes, we used the dataset GSE274532, which contains data from microglial HMC3 cells stimulated for 24 h with *P. gingivalis* strains W50, E8, and K1A at a multiplicity of infection (MOI) 10.

### Differential expression analysis

We identified differentially expressed genes (DEGs) using the GEO2R tool, which compares sample groups within a GEO Series (20). GEO2R uses a linear model to determine statistical significance, helping identify upregulated or downregulated genes and offering insights into biological processes.

### Network analysis and hub gene identification

We used Cytoscape software with the CytoHubba plugin to create gene expression networks and identify hub genes (21). CytoHubba ranks nodes in protein-protein interaction (PPI) networks based on connectivity using the MCC method. Additional tools like GeneMania were used to build co-expression networks, aiding in biomarker and therapeutic target identification.

### Prediction of interactome hub genes

For hub gene prediction, we applied logistic regression and a light gradient boosting model (LightGBM) using Python's Jupyter Notebook and the DataRobot tool. Outliers from the top 250 DEGs were removed before analysis. The dataset was split into 80% training and 20% testing sets, with 10-fold cross-validation to ensure reliable results.

### Light gradient boosting

Light Gradient Boosting (LightGBM) is a popular machine-learning method known for its high computational efficiency, memory efficiency, and accuracy. It uses a leaf-wise split approach and a histogram-based algorithm to handle large datasets and high-dimensional features. LightGBM optimizes memory usage through Gradient-based One-Side Sampling (GOSS), reducing data instances during training. It supports large-scale datasets and provides flexibility in customization, allowing users to fine-tune the model according to specific requirements. LightGBM employs a tree-based ensemble learning approach and uses gradient boosting to iteratively create an ensemble of weak prediction models called decision trees. It focuses on the leaf node with the maximum information gain in each iteration, optimizing objective functions by adding weak learners and adjusting training sample weights. Leaf-wise splitting minimizes loss function during splits, reducing tree nodes and improving performance. The dataset format allows faster training and reduces memory usage, supporting various data formats, including sparse datasets.

LightGBM creates decision tree ensembles, focusing on nodes with maximum information gain. Key parameters include:

• **num_leaves** and **max_depth**: Control tree complexity.
• **learning_rate**: Adjusts the step size.
• **n_estimators**: Defines the number of boosting iterations.
• **sample** and **sample_bytree**: Determine the fraction of data and features used.

### Logistic regression

Logistic Regression is a statistical model for classification problems, predicting the probability of an outcome belonging to a specific class. It consists of an input layer, a linear function, an activation function, a decision boundary, a loss function, an optimization algorithm, and an output layer. The input layer represents input features, which can be numerical, categorical, or combined. The linear function calculates the weighted sum of the features and adds the bias term, creating a linear boundary. The activation function transforms the linear output into a probability value between 0 and 1, with the sigmoid function being the most commonly used. The model uses an optimization algorithm to update the weights and biases iteratively, improving its predictive performance.

An optimization algorithm's parameters include the regularization strength, solver, maximum_iter, penalty, and tolerance for stopping criteria, which control the convergence threshold and prevent overfitting, ensuring optimal performance.

Predicting outcomes of the hub model involves calculating a weighted sum of features, assigning weights to each feature, and adjusting these weights during training to optimize the model's performance. The activation function, like Sigmoid, takes the linear output and transforms it into a more interpretable format. The output represents the predicted probability of the class label, indicating the confidence level of the prediction. The loss function measures how well the model's predictions match the actual outcomes, aiming to minimize this loss during training.

Logistic Regression and LightGBM are the leading algorithms for predicting outcomes related to interactomic hub genes in periodontitis and Alzheimer's Disease. Logistic Regression provides interpretability, is straightforward, and serves as a baseline for evaluating the effectiveness of more complex models. It is computationally efficient, particularly with larger datasets, and can manage multicollinearity. In contrast, LightGBM is well-known for its outstanding performance with large datasets and high-dimensional data, outpacing other gradient-boosting methods. It is designed for efficiency, enabling faster training times and improved handling of imbalanced data. Additionally, LightGBM features built-in capabilities for evaluating feature importance, providing valuable insights in biological contexts. Moreover, it offers flexibility through a variety of customizable hyperparameters. Both Logistic Regression and LightGBM effectively predict outcomes related to interactomic hub genes in periodontitis and Alzheimer's disease. They achieve a balance of accuracy, interpretability, and processing efficiency, with LightGBM utilizing a gradient boosting framework for effectively managing large datasets, while Logistic Regression is ideal for linearly related input features.

## Results

The performance metrics demonstrate the effectiveness of the Light Gradient Boosting and Logistic Regression models in predicting outcomes related to interactomic hub genes in periodontitis and Alzheimer's disease. The models achieved an accuracy of 68%, indicating moderate classification performance with potential for further optimization. The precision of 68.75% reflects a reasonable reduction in false positives, while the recall of 97.06% highlights the models' high sensitivity despite lower precision. The F1 score, approximately 0.80, indicates a balanced trade-off between precision and recall. High recall is particularly important for identifying potential genetic markers or pathways involved in disease processes, whereas precision is critical for minimizing unnecessary interventions or misdiagnoses.

Differential gene expression analysis of the validation dataset confirmed the hub genes identified in the study. Key findings include:

• **LZIC** showing the highest statistical significance,
• **TNFRSF9** demonstrating moderate significance,
• **SLC45A1**, **GPR157**, and **SLC25A33** exhibiting weak significance,
• **CD30** being absent from the dataset.

After performing differential gene expression analysis, hub genes were identified using Cytoscape with the CytoHubba plugin (21).

**Figure 1:**
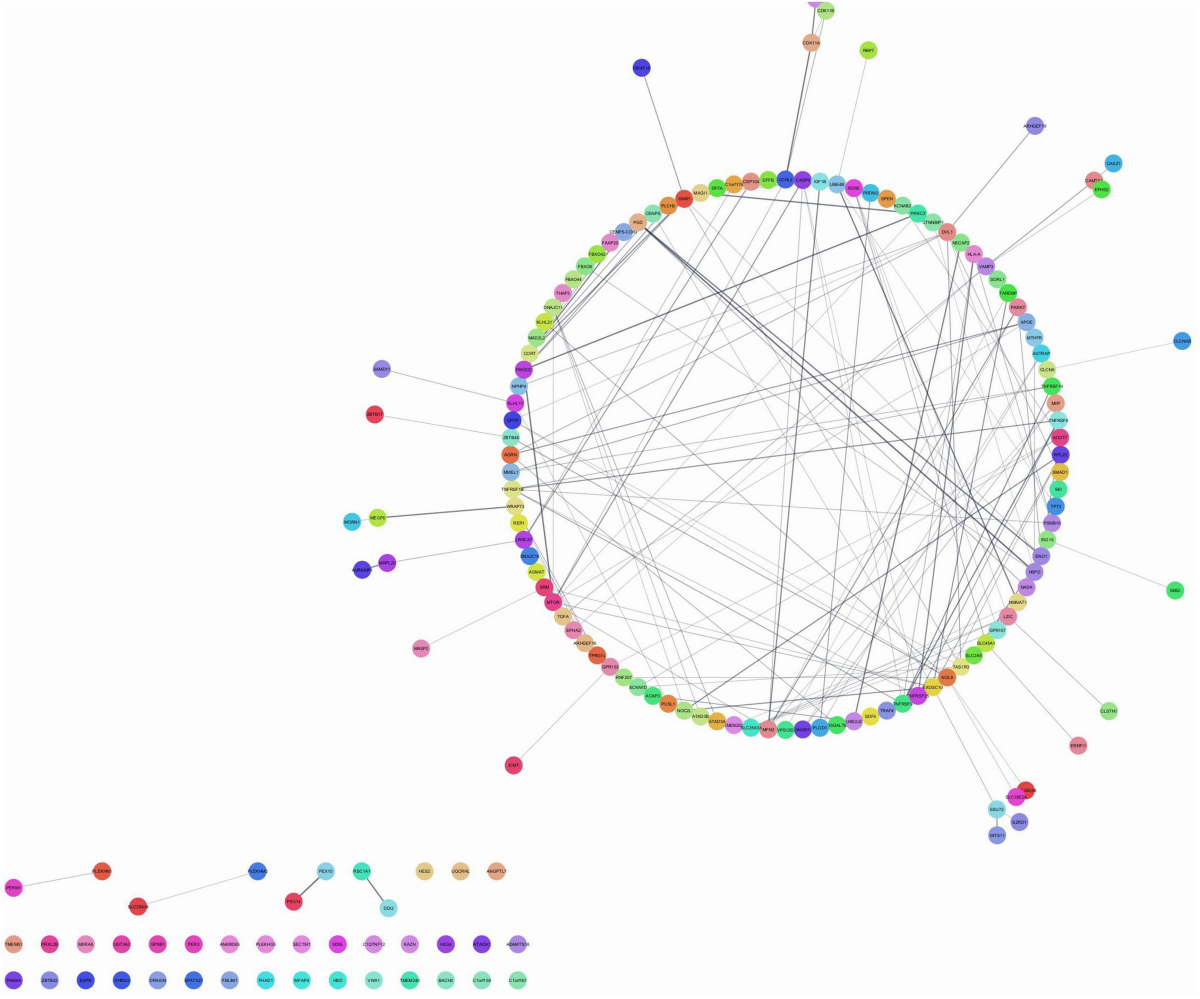
Network analysis of genes associated with endothelial dysfunction. Figure shows interactome visualization employs color-coded nodes to depict entities or data points, edges to illustrate relationships, and a circular layout to organize nodes in a circular arrangement, likely utilized to examine genes involved in endothelial dysfunction.

Figure 1 shows several nodes-174, edges-224, with neighbors-3492. The top hub genes were TNFRSF9, LZIC, TNFRSF8, SLC45A1, GPR157, and SLC25A33, induced by *P. gingivalis* and *F. nucleatum* responsible for endothelial dysfunction in brain cells. The figure describes the significance of the network analysis in understanding the genetic interactions implicated in endothelial dysfunction caused by bacterial pathogens, particularly *P. gingivalis* and *F. nucleatum*. It underscores the role of the identified hub genes as potential targets for therapeutic interventions to mitigate these pathogens' effects on brain endothelial cells.

**Figure 2:**
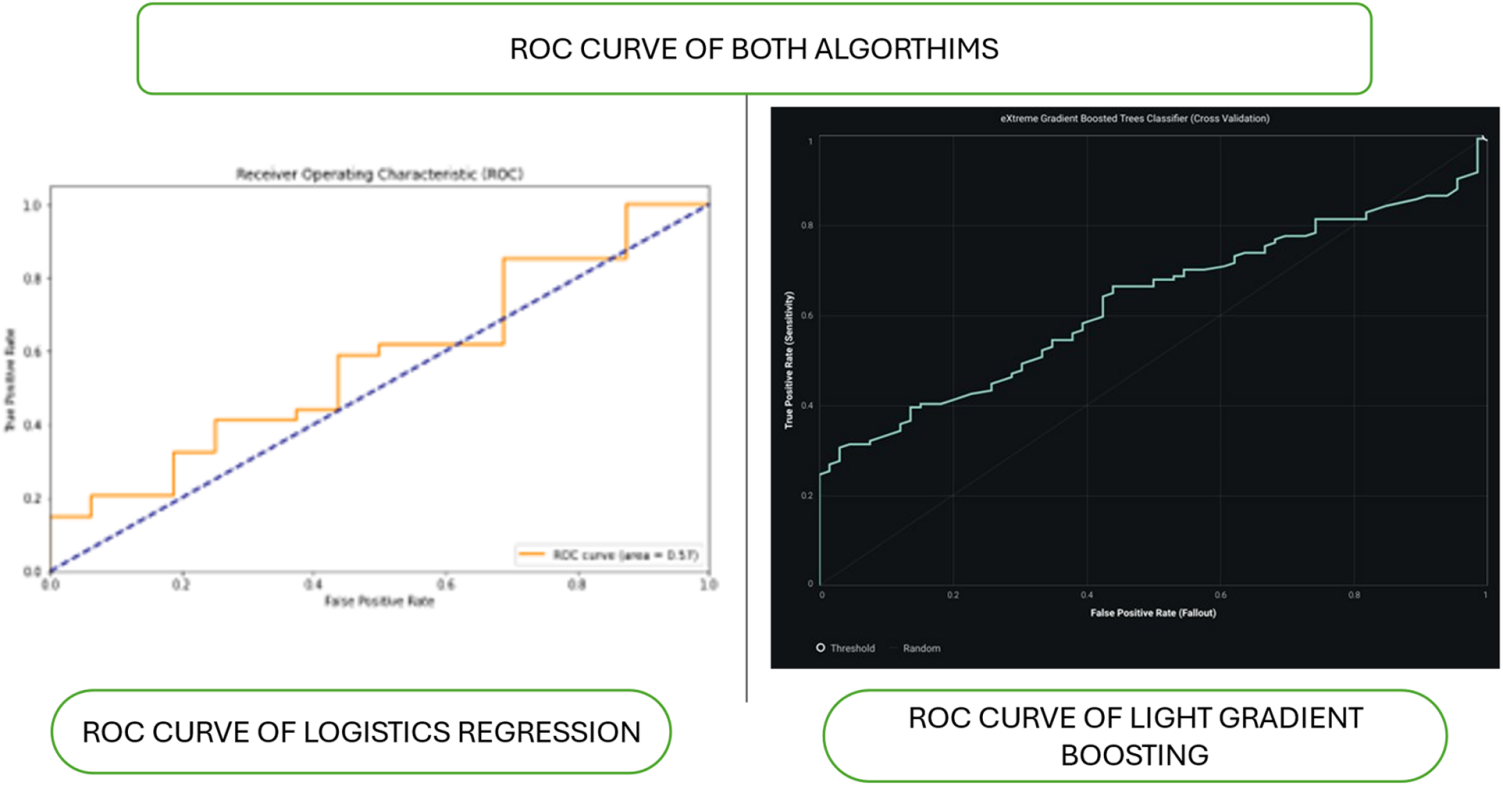
Roc curve of algorithms. Figure shows the ROC curves of two models, logistic regression and light gradient boosting, are compared. The logistic regression ROC curve, plotted with false positive rate (FPR) and true positive rate (TPR), shows poor performance with an AUC of approximately 0.5.The gradient-boosting ROC curve, which indicates better performance than logistic regression, is smoother and steeper in the right panel (light gradient boosting).

Figure 2 shows the ROC curve of algorithms. The ROC curve (Receiver Operating Characteristic) is a graphical representation of the performance of a classification model at different classification thresholds. It plots the True Positive Rate (sensitivity) against the False Positive Rate (1-specificity). The AUC (Area Under the Curve) is a metric that quantifies the model's overall performance across all possible thresholds. It ranges from 0 to 1, with 1 indicating perfect classification and 0.5 representing random guessing. The closer the AUC is to 1, the better the model accurately distinguishes between the positive and negative classes. This means the model can predict true positives more frequently while minimizing false positives. Conversely, an AUC value close to 0.5 suggests that the model is ineffective at distinguishing between the classes and that its predictions are similar to random guessing.

**Figure 3:**
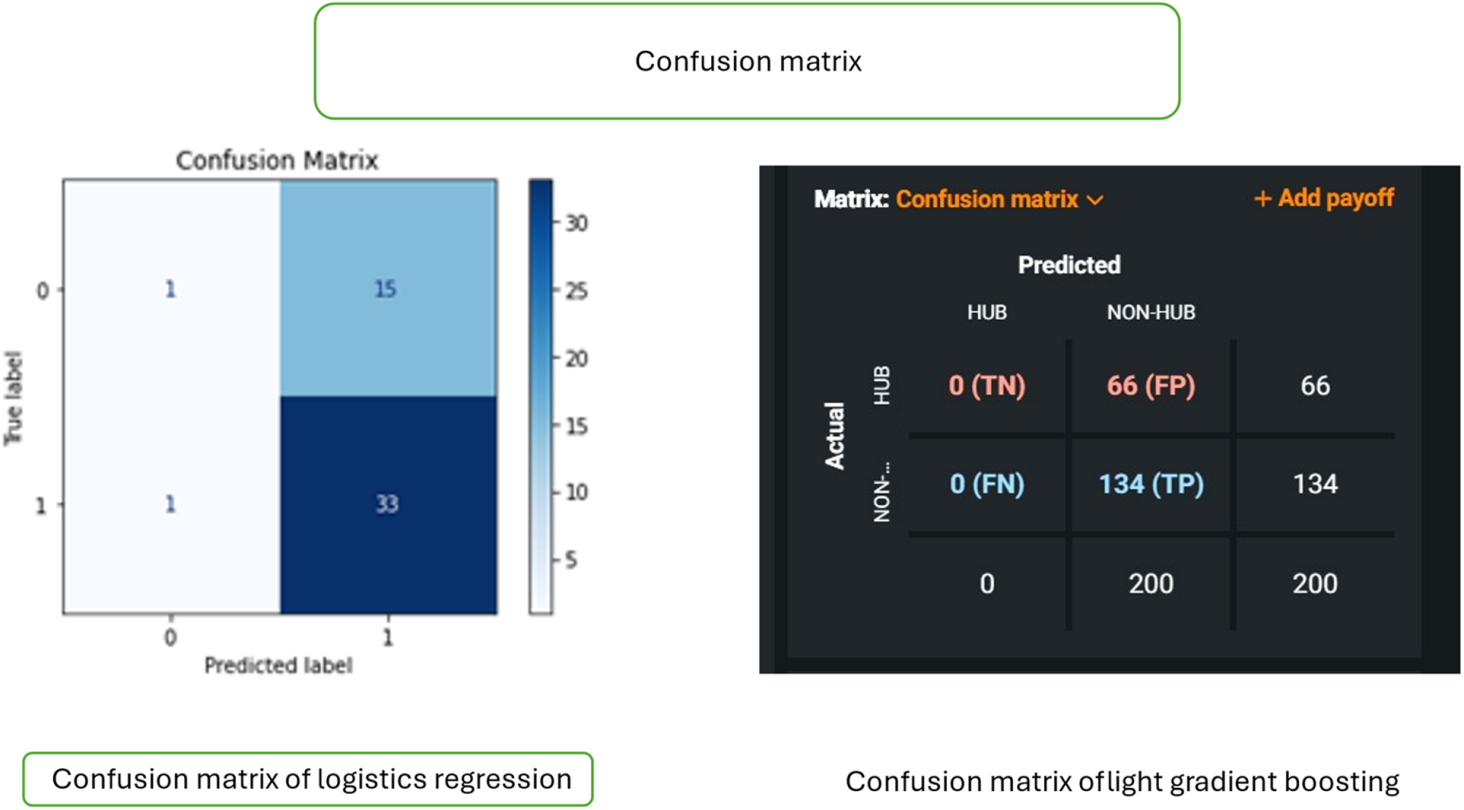
Confusion matrix comparison of logistic regression and light gradient boosting models. Figure shows the comparison of the confusion matrices of two models: logistic regression and light gradient boosting. Left panel (logistic regression): the matrix displays true labels (0, 1) and predicted labels (0, 1), including true negative (TN), false positive (FP), false negative (FN), and true positive (TP) values. Right panel (light gradient boosting): the confusion matrix illustrates true labels (*Y*-axis) and predicted labels (*X*-axis), offering insights into the classification performance of two models, with values ranging from 0 to 134.

Figure 3 shows the confusion matrix of logistics regression and light gradient boosting. The figure explains the components of the confusion matrices, including true positives (TP), true negatives (TN), false positives (FP), and false negatives (FN). It emphasizes the importance of these matrices in assessing the performance of logistic regression and LightGBM models. The figure highlights how these models contribute to predicting risk factors, guiding the choice of the optimal model for clinical applications in periodontal regeneration.

The Confusion Matrix allows for a more detailed understanding of how well the model is performing and where it might be making mistakes. It helps evaluate the model's performance in actual vs. predicted classifications. Other performance metrics, such as accuracy, precision, recall, and F1 score, can be calculated from the Confusion Matrix.

Table 1 shows the accuracy of logistic regression and light gradient boosting at 67% and 60%, respectively.

**Table 1:** Accuracy of logistics regression and light gradient boosting.

SNO	AUC	Recall	Precision	F1
Logistics regression	67%	0.97	0.68	0.80
Light gradient boosting	60%	0.74	0.78	0.81

After applying hyperparameter tuning and evaluating the logistic regression model on the testing set, the following performance metrics were obtained:

• Accuracy: 68%
• Precision: 68.75%
• Recall: 97.06%
• F1 Score: approximately 0.80

The model demonstrates a slight improvement in precision compared to the baseline, indicating a better balance between true and false positives. However, the high recall suggests that the model may be overly conservative. Overall, the model correctly predicts outcomes 68% of the time, with a precision of 68.75% and a high recall of 97.06%, resulting in an F1 score of approximately 0.80.

**Figure 4:**
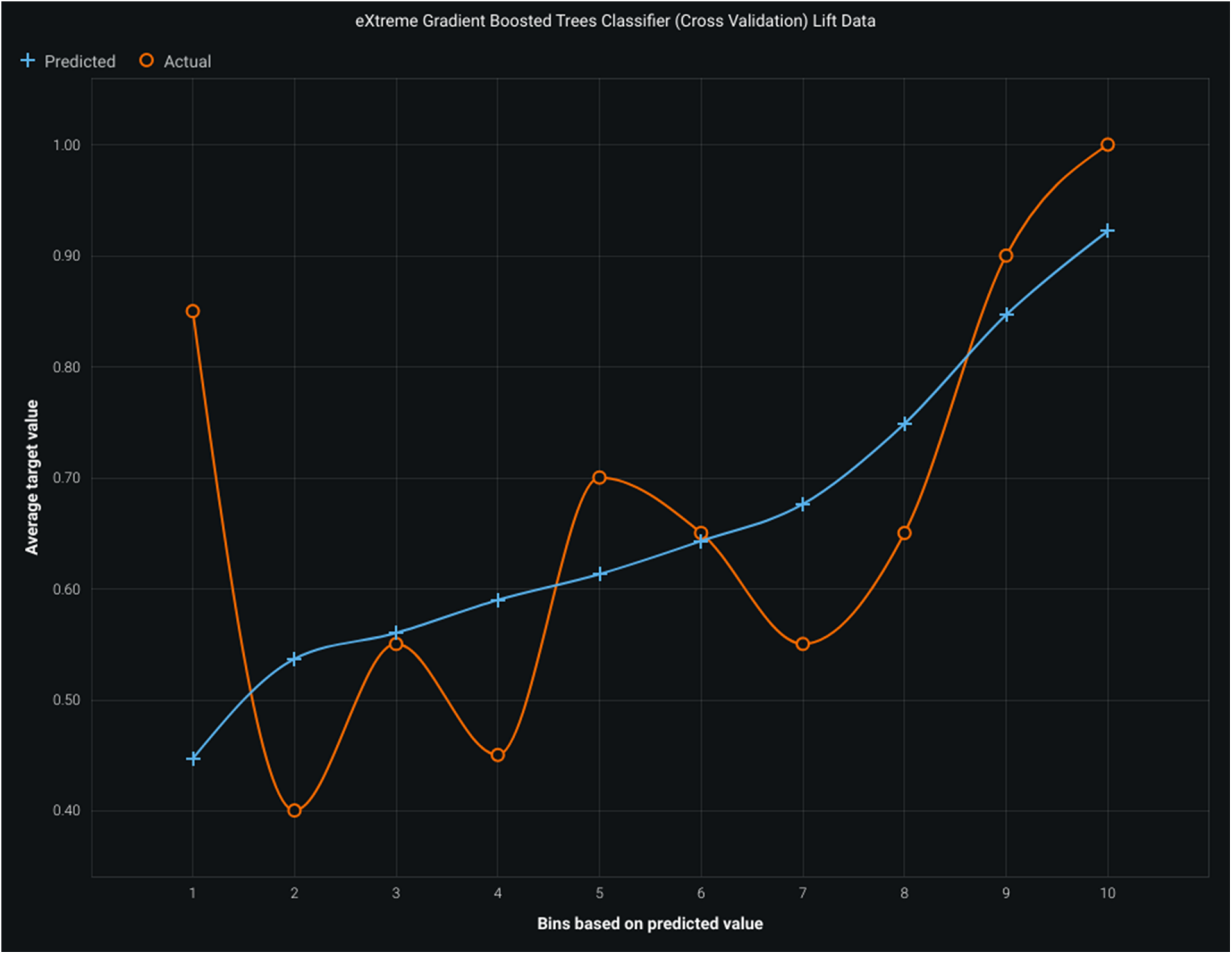
Lift chart for light gradient boosting model. Figure illustrates the bins. Based on predicted values, average target values, actual values, and the model's performance, the predicted line should closely align with the actual line, indicating an accurate prediction of the target variable.

Figure 4 shows a lift chart of light gradient boost with moderate lift showing good accuracy. A higher lift at a particular decile suggests that the model performs better in predicting the positive outcome (in this case, churn) than random selection. The optimal targeting point is often at the higher deciles, with the highest lift. The lift chart helps machine learning practitioners compare different models or algorithms and select the most effective one for the task. It also aids in determining optimal probability thresholds or cutoffs for decision-making, such as setting thresholds for customer retention efforts based on the model's predictions.

## Discussion

The oral cavity contains over 700 microorganisms, including approximately 500 bacterial species. Research highlights a positive link between periodontal disease and neurodegenerative disorders, especially cognitive decline, suggesting that periodontal pathogens could serve as targets for preventing neurodegenerative diseases (22, 23). Studies have identified elevated IgG antibodies against *A. actinomycetemcomitans*, *P. gingivalis*, and *T. forsythia* in individuals with Alzheimer's disease. Additionally, pathogens such as *P. gingivalis*, *T. denticola*, and *F. nucleatum* can infiltrate the central nervous system and trigger neuroinflammation (13, 14, 22–24). In this study, transcriptomic analysis of brain endothelial cells exposed to *P. gingivalis* and *F. nucleatum* identified key interactomic hub genes.

This study highlights several hub genes critical to the development of periodontitis. TNFRSF9 (4-1BB) regulates T-cell activation and survival, promoting inflammation and tissue damage. LZIC is involved in cell proliferation and differentiation, influencing inflammatory pathways in periodontal tissues. SLC45A1 aids in metabolite transport, while GPR157 modulates periodontal inflammation and immune responses. SLC25A33, a mitochondrial transporter, plays a role in cellular energy metabolism and may contribute to periodontal disease pathology. In Alzheimer's disease, the hub genes TNFRSF9, LZIC, CD30, SLC45A1, GPR157, and SLC25A33 are implicated in immune regulation, cellular transport, neuronal signaling, and mitochondrial function. Understanding the roles of these genes could aid in developing therapeutic strategies for disease management.

Postmortem studies detected *P. gingivalis* DNA in the cerebrospinal fluid of 8 out of 10 Alzheimer's patients, linking gingipains in the brain to Alzheimer's pathology. Furthermore, oral administration of *P. gingivalis* has been shown to activate microglial cells, increase proinflammatory cytokine production, and induce neuronal death in specific brain regions in mice, mimicking Alzheimer's features (15, 16, 25, 26). These pathological features include increased gene expression and elevated production of the Aβ1-42 peptide.

One recent study employs machine learning and deep learning to identify biomarkers for Alzheimer's Disease. It achieved an AUC of 0.979 for five specific genes using three gene expression datasets and ranking algorithms. Seventy percent of upregulated hub genes are potential AD targets, with microRNAs and JUN associated with these genes (27). Another study introduces a new method for early detection and diagnosis of Alzheimer's disease using gene selection techniques and deep learning. It uses Singular Value Decomposition and Principal Component Analysis, achieving high accuracy rates (28).

*P. gingivalis* is a well-studied periodontal pathogen strongly associated with Alzheimer's disease incidence and progression. Genome-wide association studies have revealed that host genes interacting with *P. gingivalis* are significantly linked to Alzheimer's disease. Its DNA and virulence factors have been detected in Alzheimer's patients' brains, inducing Alzheimer-like pathology in mice. Prior studies using the Boruta algorithm identified 48 crosstalk genes between periodontitis and Alzheimer's, including C4A, C4B, CXCL12, FCGR3A, IL1B, and MMP3 (29, 30). Similarly, our study revealed genes like TNFRSF9, LZIC, TNFRSF8, SLC45A1, GPR157, and SLC25A33 that contribute to endothelial dysfunction in brain cells due to *P. gingivalis* and *F. nucleatum*.

Previous studies (13, 15, 24, 31–35) have utilized immunocorrelation analysis to identify immune-related genes, cells, and pathways involved in both AD and periodontal disease (PD). These studies revealed that M2 macrophages and NKT cells are highly expressed in both conditions, suggesting potential involvement of these immune cells in the pathogenesis of both diseases. B-cells, CD4 + memory T-cells, and CD8 + naive T-cells were also found to be elevated in both AD and PD, indicating a potential role for infiltrating immune cells in disease progression. These findings highlight the importance of immune responses in the development and progression of both AD and PD.

The logistic regression model demonstrated superior performance compared to the light gradient boosting model in predicting interactomic hub genes, achieving an accuracy of 67% compared to 60%, respectively (Figures 1–4; Table 1). The logistic regression model exhibited high sensitivity (97.06%), indicating its ability to accurately identify true positives. The model also demonstrated a balanced performance with an F1 score of approximately 0.80, indicating a good balance between precision and recall. The low false positive rate of 68.75% further enhances the clinical applicability of the model. These findings are comparable to previous studies using machine learning models, such as the random forest model, which achieved high accuracy in predicting human-spike and drug-protein interactions (36).

This study highlights the strengths of machine learning models, such as Logistic Regression and Light Gradient Boosting, in predicting interactomic hub genes associated with periodontitis and Alzheimer's Disease. However, it is important to acknowledge the limitations of these models. Data quality and sample size can significantly impact model performance and generalizability. The effectiveness of the models depends on the selection and quality of the features used for training. Furthermore, interpreting the biological significance of the model's predictions can be complex. Additionally, the models may exhibit biases towards more prevalent classes, potentially leading to skewed performance metrics.

This study examines the roles of *P. gingivalis* and *F. nucleatum* in periodontitis and Alzheimer's disease. Confounding factors include comorbidities, age, genetics, environmental influences, sample size and diversity, and methodological differences. Understanding these factors can enhance the validity and reliability of findings, leading to more robust conclusions about the involvement of specific pathogens in the development of Alzheimer's disease. Addressing these variables can result in more precise therapeutic interventions and better outcomes in both periodontal and cognitive health.

## Conclusion

The logistic regression model showed better overall performance than the light gradient boosting model in predicting hub genes. Both models can be further improved to enhance their accuracy and applicability in predicting hub genes associated with periodontal disease and Alzheimer's disease.

## Data Availability

Publicly available datasets were analyzed in this study. This data can be found here: NCBI GEO: archive for functional genomics data sets-update. Nucleic Acids Res. 2013 Jan; 41(Database issue): D991-5.
